# Supplementary material for: Parents and GPs’ understandings and beliefs about food allergy testing in children with eczema: qualitative interview study within the Trial of Eczema allergy Screening Tests (TEST) feasibility trial
Source: BMJ Open. 2020 Nov 18;10(11):e041229. doi: 10.1136/bmjopen-2020-041229 (PMC7677338; doi:10.1136/bmjopen-2020-041229)
Supplement: Supplementary data [file bmjopen-2020-041229supp001.pdf]

TEST Interview Topic Guide – PARTICIPATING PARENT

1. *Introduction*

- Thank the interviewee, introduce self, re-state purpose of the interview and main points from the information sheet, answer any questions, outline structure of interview
- Discussion of how interview will be recorded and transcribed, right to withdrawal from interview, issues of confidentiality, anonymisation and informed consent – *if agree start audio recorder*
- Verbal consent
    - Do you agree to our conversation being audio recorded?
    - Do you know you are free to stop the interview at any point and you may skip questions you would prefer not to answer?
    - Do you understand that quotations from the interview may be used to illustrate our findings, but it will not be possible to trace who said them?

2. *Background*

- Age of child and general eczema history: how/when was eczema first diagnosed? Length time eczema been an issue, severity over time
- Explore eczema management: Where have they sought advice on managing eczema? Doctor, health nurse, alternative practitioner (outside NHS), internet including forums? Any investigations up to point of study? What treatments advised/tried to date?
- How many children? Do siblings have eczema? What about related conditions e.g. asthma, hay fever?
- Currently breastfeeding? Y/N

3. *Beliefs about allergy testing and role of allergies in eczema*

- What do they know about the origins/cause of eczema?
- Do they think their child's eczema is related to an allergy (food or other)? Why do they give the answer they do?
- What does the parent know about food allergies? Is the parent aware of distinctions between food allergy, food intolerance, local skin irritation?
- Have they sought advice about food allergy testing? Explore understanding of food allergy testing e.g. different types of tests, food exclusion
- Explore previous experiences of food allergy testing in the family:
    - Have their child/other family members had allergy testing or excluded certain foods? If yes, how/why did this come about? Where did they seek advice on allergy testing/food exclusion?
    - What sort of tests - skin prick test, blood test? Other forms of testing encountered? Was this at home or through the NHS/private (and reason for this)? *Note: child should not have had food allergy testing other than at home as inclusion criterion*
- In their opinion, why would any parent want their child to have a food allergy test? Why would any parent not want their child to have food allergy test? Explore views of both food elimination and reintroduction strategy and skin prick tests. Are there reasons specific to skin prick tests?
- What about them – would they allow their child to have it or not? Probe reasons why it is/is not acceptable (generally, and skin prick test more specifically)

- What does parent think about excluding certain foods from the child's diet? What might challenges be (if any)? Worry? Social difficulties (parties, eating out)? School? Family set up? Shared parenting? Is there anything which could be done to overcome challenges?

4. *Views on the trial and experiences of participating*

- How did you first hear about the study? (letter, approached HCP, poster, flyer etc.)
- Views on being asked to take part in the study. Initial thoughts? What did they think about initial invitation letter, flyer, name of study/logo? PIS, baseline visit?
- Understanding of the purpose of trial. Understanding of what's involved in taking part in the trial. What does the parent like about the study?
- Why did the parent choose to take part in study (motivations)? Any concerns? Have these been overcome? How, why?
- Breastfeeding. What advice do you/would you want about your own and the child's diet? Any concerns?

Select which of the below sets of questions to ask depending on whether shorter/longer time since randomisation:

- **Early in trial (shorter time since randomisation)**
  - Clarify with route taken since allocation to trial arm. What's happened, who have they been referred to?
  - Parent's expectations
  - Understanding of procedures/what they need(ed) to do
  - What are their views on what happens next?
  - What does the parent expect to happen next? Explore expectations of skin prick test and food avoidance (if intervention)
  - Does the parent anticipate sticking to which arm they have been allocated? Why/why not?
  - Is there anything the parent would change about the way they have been involved in the trial so far?
  - What is their experience of completing the study questionnaires? Probe length, ease of completion, difficulty understanding/answering any items, burden
  - Check whether skin prick test done at same time as allergy history questionnaire. If yes, does the parent think their answers to the allergy history were influenced by having the skin prick test done at same time?
  - Has the parent spoken with other parents about being involved in the trial? Explore answer

- **Later in trial (longer time since randomisation)**
  - Clarify route taken since allocation to trial arm. What's happened, who have they been referred to?
  - Explore experiences of visits and tests, and their opinion of these:
    - How understandable was the advice?
    - Was it presented in a format they wanted it in?
    - Could they remember what they had been advised?
    - Explore views of information they may have been given in between
  - Was parent happy with outcomes of visits, tests, treatment, control allocation. Any concerns?
  - Experiences of eczema management since allocation. What has the parent been doing? Is this in line with what was recommended as part of trial allocation (i.e. did they stick to what was recommended)? What has gone well? Have there been any challenges? Do you think you are likely to continue to follow the advice?

- Understandings of components of intervention – skin prick test and oral food challenge:
  - Any concerns? What did the parent expect to happen? Did what happen match expectations? If not, what were the differences? Any unexpected effects?
  - Was the information provided about the test/challenge adequate or not? Was anything missing?
  - How seriously did they take the results of the tests? Why? Did the test results lead to certain actions/behaviours? If child is an infant, were there any changes to breast-feeding practices because of the intervention?
- Have there been any changes in their attitudes and beliefs about allergy testing? If yes, what changes and why? What aspects of what they experienced made most difference? Probe: written information (which topics – emollients, food allergies?), verbal explanations from dietician, website, skin prick test etc
- Is there anything the parent would change about the way they have been involved in the trial?
- What was their experience of completing the study questionnaires? Probe length, ease of completion, difficulty understanding/answering any items, burden
- Has the parent spoken with other parents about being involved in the trial and the skin allergy testing? Explore answer
- How long they would be prepared to keep feeding back to the trial?

5. Any other issues

- Any other issues the participant would like to raise?
