# Supplementary material for: Parents and GPs’ understandings and beliefs about food allergy testing in children with eczema: qualitative interview study within the Trial of Eczema allergy Screening Tests (TEST) feasibility trial
Source: BMJ Open. 2020 Nov 18;10(11):e041229. doi: 10.1136/bmjopen-2020-041229 (PMC7677338; doi:10.1136/bmjopen-2020-041229)
Supplement: Author's manuscript [file bmjopen-2020-041229.draft_revisions.pdf]

BMJ Open

BMJ Open is committed to open peer review. As part of this commitment we make the peer review history of every article we publish publicly available.

When an article is published we post the peer reviewers' comments and the authors' responses online. We also post the versions of the paper that were used during peer review. These are the versions that the peer review comments apply to.

The versions of the paper that follow are the versions that were submitted during the peer review process. They are not the versions of record or the final published versions. They should not be cited or distributed as the published version of this manuscript.

BMJ Open is an open access journal and the full, final, typeset and author-corrected version of record of the manuscript is available on our site with no access controls, subscription charges or pay-per-view fees (http://bmjopen.bmj.com).

If you have any questions on BMJ Open's open peer review process please email info.bmjopen@bmj.com

BMJ Open

Parents and GPs' understandings and beliefs about food allergy testing in children with eczema: qualitative interview study within the Trial of Eczema allergy Screening Tests (TEST) feasibility trial

Journal:	*BMJ Open*
Manuscript ID	bmjopen-2020-041229
Article Type:	Original research
Date Submitted by the Author:	03-Jun-2020

Complete List of Authors:	Clement, Clare; University of Bristol, Bristol Medical School<br>Ridd, Matthew; University of Bristol Faculty of Health Sciences, Population Health Sciences;<br>Roberts, Kirsty; University of Bristol School of Social and Community Medicine<br>Santer, Miriam; University of Southampton, Primary Care & Population Sciences<br>Boyle, R; Imperial College London; University of Nottingham<br>Muller, Ingrid; University of Southampton, Department of Psychology<br>Gilbertson, Anna; University of Bristol, School Of Social & Community Medicine<br>Angier, Elizabeth; University of Southampton, Primary Care & Population Sciences<br>Selman, Lucy; University of Bristol School of Social and Community Medicine<br>Shaw, Ali; University of Bristol Faculty of Arts, Bristol Medical School

Keywords:	Eczema < DERMATOLOGY, NUTRITION & DIETETICS, PRIMARY CARE, Allergy < THORACIC MEDICINE, QUALITATIVE RESEARCH, Community child health < PAEDIATRICS

SCHOLARONE™
Manuscripts

I, the Submitting Author has the right to grant and does grant on behalf of all authors of the Work (as defined in the below author licence), an exclusive licence and/or a non-exclusive licence for contributions from authors who are: i) UK Crown employees; ii) where BMJ has agreed a CC-BY licence shall apply, and/or iii) in accordance with the terms applicable for US Federal Government officers or employees acting as part of their official duties; on a worldwide, perpetual, irrevocable, royalty-free basis to BMJ Publishing Group Ltd ("BMJ") its licensees and where the relevant Journal is co-owned by BMJ to the co-owners of the Journal, to publish the Work in this journal and any other BMJ products and to exploit all rights, as set out in our licence.

The Submitting Author accepts and understands that any supply made under these terms is made by BMJ to the Submitting Author unless you are acting as an employee on behalf of your employer or a postgraduate student of an affiliated institution which is paying any applicable article publishing charge ("APC") for Open Access articles. Where the Submitting Author wishes to make the Work available on an Open Access basis (and intends to pay the relevant APC), the terms of reuse of such Open Access shall be governed by a Creative Commons licence – details of these licences and which Creative Commons licence will apply to this Work are set out in our licence referred to above.

Other than as permitted in any relevant BMJ Author's Self Archiving Policies, I confirm this Work has not been accepted for publication elsewhere, is not being considered for publication elsewhere and does not duplicate material already published. I confirm all authors consent to publication of this Work and authorise the granting of this licence.

For peer review only

Parents and GPs' understandings and beliefs about food allergy testing in children with eczema: qualitative interview study within the Trial of Eczema allergy Screening Tests (TEST) feasibility trial

Authors:

Clare Clement MSc[1], Matthew J Ridd PhD[2], Kirsty Roberts PhD[2], Miriam Santer PhD[3], Robert J Boyle PhD[4, 5], Ingrid Muller PhD[3], Anna Gilbertson MSc[2], Elizabeth Angier MSc[3], Lucy Selman PhD[1,2], Alison R G Shaw PhD[2]

[1] Bristol Trials Centre, Bristol Medical School, University of Bristol, Bristol, UK

[2] Population Health Sciences, University of Bristol, UK

[3] Primary Care, Population Sciences & Medical Education, University of Southampton, Southampton, UK

[4] Imperial College London, London, UK

[5] Centre of Evidence-Based Dermatology, University of Nottingham, Nottingham, UK

Corresponding author:

Clare Clement, Bristol Trials Centre, Bristol Medical School, University of Bristol, Bristol, UK.
c.clement@bristol.ac.uk, (0117) 9287269

Keywords

Eczema, parents, diet, child, primary care practice, allergens, qualitative

Word Count: 3999

For peer review only - http://bmjopen.bmj.com/site/about/guidelines.xhtml

ABSTRACT

Aim: To explore parent and General Practitioner (GP) understanding and beliefs about food allergy testing for children with eczema.

Design and Setting: Qualitative interview study in UK primary care within the Trial of Eczema allergy Screening Tests (TEST) feasibility trial.

Participants: Semi-structured interviews with parents of children with eczema taking part in the feasibility study and GPs at practices hosting the study.

Results: 21 parents and 11 GPs were interviewed. Parents discussed a range of potential causes for eczema, including a role for food allergy. They believed allergy testing to be beneficial as it could potentially identify a cure or help reduce symptoms and they found negative tests reassuring, suggesting to them that no dietary changes were needed. GPs reported limited experience and uncertainty regarding food allergy in children with eczema. While some GPs believed referral for allergy testing could be appropriate, most were unclear about its utility. They thought it should be reserved for children with severe eczema or complex problems but wanted more information to advise parents and help guide decision-making.

Conclusions: Parents' motivations for allergy testing are driven by the desire to improve their child's condition and exclude food allergy as a possible cause of symptoms. GPs are uncertain about the role of allergy testing and want more information about its usefulness to support parents and help inform decision making.

Trial registration: ISRCTN15397185

For peer review only - http://bmjopen.bmj.com/site/about/guidelines.xhtml

STRENGTHS AND LIMITATIONS OF THIS STUDY

- We believe this is the first qualitative study to specifically explore GPs' and parents' views regarding the role of food allergy in children with eczema.
- We interviewed GPs and parents with a range of characteristics and employed a topic guide flexibly to ensure that different aspects of food allergy and testing in children with eczema were captured.
- By virtue of taking part in the trial, to some extent all participants were open to the idea of children with eczema undergoing Skin Prick Tests for food allergies.
- Other healthcare professionals and less well-educated parents may have different experiences and opinions.

For peer review only

For peer review only - http://bmjopen.bmj.com/site/about/guidelines.xhtml

INTRODUCTION

Eczema (synonyms atopic eczema/dermatitis) is a common and burdensome condition, especially among pre-school age children. (1) Clinical guidelines emphasise the importance of avoiding environmental irritants and practising good skin care through regular use of emollients and appropriate use of topical corticosteroids. (2)

A concern among parents of children with eczema, voiced to GPs and commonly seen in online forums, is the role of food allergy. (3-5) Despite weak evidence to support dietary modification, many parents try excluding foods from their child's diet to reduce eczema symptoms or the need for treatment with medications. (6, 7)

In the UK, NICE guidance (2, 8) recommends that healthcare professionals consider food allergy as potential triggers in children with eczema if they develop symptoms immediately after ingesting a potential allergen, or in those with moderate to severe eczema who have not responded to optimum management. Immediate-type food allergies are more common in children with eczema (9, 10) and food allergy testing may potentially prevent serious allergic reactions and/or identify foods causing eczema symptoms. However, in primary care professional advice as to the importance of allergy testing for children with eczema is variable, as is access to allergy testing. (11) In principle, if allergy testing were shown to inform eczema care then it could be routinely offered in primary care. However, the effectiveness of food allergy testing and associated dietary measures for managing eczema is uncertain.

In addition, there is also uncertainty about the feasibility and acceptability of conducting research to answer the question of if, or when, food allergy testing should be routinely offered to children with eczema. It is important to resolve these uncertainties to help inform parents and GPs decision making about optimising diet and management of eczema.

Nested within the feasibility Trial of Eczema allergy Screening Tests Study (TEST) study, (12) we report on a qualitative study that explored parent and GP understanding and beliefs about food allergy testing for children with eczema.

For peer review only - http://bmjopen.bmj.com/site/about/guidelines.xhtml

METHOD

Study design

The TEST study was conducted to determine the feasibility of conducting a trial comparing test-guided dietary management versus usual care, for the management of eczema in children. More detail can be found elsewhere, (12) but in brief it was a single centre, two-group, individually randomised, feasibility randomised controlled trial conducted in 17 GP surgeries in the West of England. Children aged between >3 months and <5 years with mild or worse eczema were randomised to either control (usual care) or intervention. The intervention comprised a structured allergy history and Skin Prick Tests (SPTs) for cow's milk, hen's egg, wheat, peanut, cashew, and codfish. Dietary advice was given, based on test results, to continue eating/introduce as normal or to try excluding and reintroducing one or more foods from the child's diet. Where appropriate, referral was made for an oral food challenge, where the child is exposed to a potential allergen under supervision, (12) or to a local allergy clinical for review. All participants were followed-up for six months.

Semi-structured interviews were conducted with a sample of parents of children in the TEST trial and GPs from participating practices. The aim was to explore participants' beliefs about the role of food allergy in children with eczema, the acceptability of testing and potential barriers to and facilitators of the uptake of food allergy testing in primary care. Data regarding experience of study-specific trial processes are reported elsewhere. (Ridd et al. Test-guided dietary management in children with eczema: a randomised, controlled feasibility trial (TEST)).

Sampling and recruitment

Purposive sampling was used to capture maximum variation in views and experiences. Parents were sampled from both the intervention and usual care groups and reflected mild/moderate (<17) vs severe (≥17) Patient Orientated Eczema Measure (POEM) symptom score, (13) and socio-economic status (assessed using the Index of Multiple Deprivation Decile (IMDD) (categories: high (8-10)/ medium (5-7)/low (1-4)) (14). Participants allocated to the intervention group were also sampled based on whether they had a negative or positive SPT results. The sampling of GPs captured variation in IMDD (14) of the practice postcode and doctor characteristics (length of time practicing as a GP and self-reported confidence in managing eczema (scale 1-10, 1=low, 10=high). Sample size was informed by the team's judgement that we had enough "information power" to meet the study aims. (15)

For peer review only - http://bmjopen.bmj.com/site/about/guidelines.xhtml

Data collection

Interviews were conducted face-to-face and via telephone by CC (17 parents), an experienced social science researcher and KR (4 parents), the Trial Manager with experience of qualitative research. Written consent was obtained for the face-to-face interviews and verbal consent was recorded for the telephone interviews. Interviews lasted between 16 and 43 minutes (mean 25 minutes). No notable differences in length or depth of data were seen between face-to-face and telephone interviews. A flexible, semi-structured topic guide was used to assist questioning but allow participants to introduce and discuss new issues (Box 1).

Data analysis

Interviews were audio-recorded, transcribed verbatim, anonymised, and imported to Nvivo10 (16) for data management and coding. Analysis started shortly after data collection started and analytical insights were fed back into further data collection and analysis. Transcripts were analysed thematically using both inductive and deductive coding. (17) Transcripts were coded to establish an initial coding framework and study team members (CC, ARGS, KR and MJR) each independently coded a sub-set of seven transcripts; any discrepancies were discussed to ensure a coding consensus and maximise rigour. (18) The framework was then applied to all the remaining transcripts by CC. Emergent findings were discussed in regular multi-disciplinary Trial Management Group meetings to enhance validity. After coding was completed we drew on the Common-Sense Model to help interpret findings. (19)

Patient and Public Involvement (PPI)

Two parents of children with eczema were members of the Trial Management Group and advised on qualitative data collection and analysis. In addition, PPI feedback was the incorporated into the final topic guide.

RESULTS

Twenty-one parents were interviewed from eleven trial practices (Table 1). Eleven GPs were interviewed from seven trial practices (Table 2). Four main themes emerged from the analysis: *Parents' causes and associations, Knowledge and awareness, Searching for a 'cure' or seeking reassurance around current dietary practice,* and *Parents' responses to food allergy test results*.

For peer review only - http://bmjopen.bmj.com/site/about/guidelines.xhtml

Parents' causes and associations

Parents seemed unsure of the causes of eczema and discussed several possible factors. They received advice and information from a wide range of sources such as other parents, doctors, family members and the media. Some parents acted on this information, including by removing foods from their child's diet.

Parents believed that family history, age or environmental factors were responsible for their child's eczema, with some discussing how factors could interact or vary depending on the individual.

> *"I think it must be like a heat thing…it was just to see if something that is genetic that my wife had when she was little and then she grew out of it…But that's really everything we know… But I do have a lot of allergies, so I don't know if she's inherited it." (Parent 1)*

> *"To be honest I don't know an awful lot…I think that obviously dry skin, like water does affect it, but what actually causes it I don't know whether its stuff in the environment…I think there are different causes for different people as well." (Parent 7)*

Parents reported being influenced by media coverage of eczema and anecdotal stories from other parents whose child's symptoms improved when certain foods, particularly dairy, were eliminated from their diet.

> *"I've got a friend that her little girl…was actually allergic to dairy so that resolved some of her skin problems…and so being told by a friend actually dairy's not good for them and so you prevent them eating certain things…and that will resolve the problem." (Parent 9)*

Some parents had been alerted to the possible role of food allergies by speaking with health care professionals, including GPs and health visitors, about eczema, who had suggested trying elimination diets or keeping a food diary.

> *"That's what the doctor told us anyway, they said we might need to consider food allergies…they did say that if babies already have allergies, say like a milk allergy, then certain things can be a problem…I think they mentioned soya as well…they said a food diary would be a good idea." (Parent 2)*

For peer review only - http://bmjopen.bmj.com/site/about/guidelines.xhtml

"The health visitor proposed, she suggested that, to run an elimination diet." (Parent 21)

Several parents had explored the role of food in their own child's eczema, before taking part in TEST. In most parent accounts this did not improve the child's condition or improvement was not clearly linked to the change in diet.

"It may be food…so we did quite a lot of changing things at the time…we tried keeping a food diary…I think we started off offering a new thing every couple of days just to see if she reacted to it…there was nothing which obviously made her significantly worse….we thought maybe eggs but then we reintroduced them…and she was fine." (Parent 2)

"Whether it was a natural improvement, or it was the milk-free diet, I can't say but that's what happened at that point." (Parent 21)

Some parents, although aware of such stories, were not convinced and wanted more 'evidence' before they acted.

"Probably someone has said to me have you tried cutting out dairy and I haven't…If I actually listen to people with their anecdotal things or…my friend's child had eczema and they stopped them having dairy, but I'm a little bit cynical." (Parent 7)

"Everyone always says oh there's a link between dairy and eczema, but we could never really find any studies or like have it proven…there's a lot of hearsay but it hasn't actually been proven." (Parent 10)

Some parents believed food allergy may be a factor in their child's eczema by observing a link between their child eating certain foods and their eczema getting worse or eczema occurring when foods were introduced. Others did not see a role or reported having independently "ruled it out" through exclusion of certain foods from their child's diet.

"I used to find if she drank certain things…she would start to scratch and then her eczema started to flare up. Sometimes I would notice after she'd eaten chocolate or something she'd flare up as well." (Parent 3)

"I did strongly think it was to do with foods because of when I stopped breastfeeding it was something that flared up." (Parent 12)

For peer review only - http://bmjopen.bmj.com/site/about/guidelines.xhtml

"I tried cow's milk, all fish, banana…and nothing, no changes…It's not food. I'm sure it's not food." (Parent 17)

Knowledge and awareness

Knowledge and awareness of food allergy and food allergy testing and its role in managing eczema varied across both parents and GPs. Some parents were aware of what food allergies were before the trial and named common allergens, with some reporting personal experience of such food allergies. However, some parents still reported limited knowledge and understanding of food allergies.

"I know there's loads of them. The main ones are dairy and gluten and nuts. That's probably it as far as my knowledge goes." (Parent 17)

"I'm not really clued up on much of it…I have certain foods myself my tongue flares up…but other than that I don't really have any clue of food allergies." (Parent 12)

Parents and GPs labelled a food allergy as a set of acute or severe symptoms which could arise from a potential reaction to food. Symptoms which were perceived to be less severe, delayed or gastrointestinal upset were labelled as an intolerance rather than allergy.

"So I would say true allergy causes an allergic reaction, so causes an ideated reaction, so usually presents as sort of problems with breathing, lip swelling, skin response so like hives, urticaria, whereas I think intolerance seems to come with GI upset or sometimes can have skin reactions but tends not to create a full blown allergic response." (GP 9)

"I think an intolerance [is] just where it might upset them a little bit, you might get mild stomach cramps… whereas an allergy where they mouth might swell up or affect their breathing…more severe." (Parent 11)

Parents who had asked about allergy testing reported frustration with responses from GPs who believed the child did not have severe enough eczema to warrant testing.

"I asked if he could have allergy testing just to make sure it wasn't anything like that, but they said they don't tend to do it in young children unless it's [eczema] like severe." (Parent 8)

For peer review only - http://bmjopen.bmj.com/site/about/guidelines.xhtml

One parent commented on how they felt that GPs did not have the appropriate information to advise parents:

> *"I don't think there's any solid research to say whether or not there is a link so…it's difficult for doctors to advise on something there isn't any evidence for." (Parent 8)*

GPs reported limited experience of and knowledge about food allergy in general and wanted more information to guide their decision making for allergy testing in children with eczema.

> *"[A] minefield is how I would put it, I think. It's not something we're taught well at medical school…there's a massive online presence about allergy testing, much of it not evidence based. So, there's a big unmet need with parents coming with questions and I think doctors have an unmet educational need." (GP 5)*

GPs reported parents frequently requesting food allergy tests for their children. Some more inexperienced GPs reported being uncertain about the evidence and found advising parents difficult. Few of the GPs had referred a child with eczema specifically for food allergy tests, preferring to refer them to a dermatologist for more general advice or telling parents to keep a food diary.

> *"I don't feel completely confident in knowing its [allergy testing] limitations and uses…we get parents requesting allergy testing and it sort of feels like my training has always suggest there isn't necessarily a role in most cases, but there might be a role in some cases, so I'm not completely clear about the evidence behind it so I find it a bit tricky to advise parents on that." (GP 3)*

Search for a 'cure' or seeking reassurance around current dietary practice

Parents' motivation and willingness for their child to have food allergy testing was influenced by a range of factors. While parents were uncertain of the role of food allergy in eczema, they discussed how food allergy testing might be beneficial in identifying a cause for their child's eczema, providing a cure or helping with management of the condition.

> *"I just want to get on top of it [eczema] but you just can't because you don't know what is triggering it off…just to rule it [food allergy] out…then I can just stop him from having that food and then it won't bother him" (Parent 5)*

For peer review only - http://bmjopen.bmj.com/site/about/guidelines.xhtml

For peer review only

They also appeared to seek reassurance or support for their current dietary management strategy; for example, they wanted to know they were doing "the right thing" for their child by excluding or not excluding foods:

> *"I'd really like to know if, because I've had so many people say to me about dairy and 'cos I haven't done it, so I'd like to know that I'm doing the right thing."* (Parent 7)

However, some parents did express concerns about the impact that identifying a food allergy could have on their family. There were concerns for balanced nutrition and difficulties accommodating different diets within families:

> *"You need to make sure that they're getting enough fats, particularly for children and protein and fibre and everything else and carbohydrates when you're excluding all of this, and it could be actually quite difficult for some parents, some parents might have other children so the other children are eating one thing and…have to do it for the whole household." (Parent 18)*

Some parents were concerned the skin prick test could be uncomfortable for their child and were relieved their child was in the usual care group and did not have it.

> *"I was quite glad then to be honest he didn't have to do allergy testing…once I saw the fact that he wasn't that happy about being probed and prodded anyway I thought yeah, probably better off not having to do it." (Parent 7)*

Some GPs said they believed allergy testing to be appropriate for some children with eczema as it could be useful for informing potential dietary alterations to help manage the eczema. But most GPs, particularly those with more experience, had reservations about the usefulness of testing and were cautious about making referrals to allergy clinics. GPs reported being more likely to refer children for food allergy testing in severe or complex cases and where the cause of the eczema was not clear, and said they were often guided by parental wishes:

> *"I tend to discourage it [allergy testing] if I'm honest…I think unless we're having problems getting a child's eczema and their symptoms under control…I think in a child who would be very severely affected I would because I think well we're not getting this under control, we need more information…if there was a family history of food intolerance, allergies, those sort of situations." (GP 6)*

For peer review only - http://bmjopen.bmj.com/site/about/guidelines.xhtml

Whether parents or GPs thought the child may grow out of the eczema also influenced decisions to consider food allergy testing.

"Most children grow out of it and most of the time it's quite mild I think that most of the time people just tend to treat it and not perhaps think about the allergy side of things." (GP 1)

Parents' responses to food allergy test results

Parents in the intervention group expressed a range of responses to the food allergy test results. Most parents had faith in the healthcare professionals and the test and therefore accepted the results as being accurate.

"I couldn't see any reason not to trust it…I'm pretty trusting in professionals." (Parent 16)

Most results were negative, but parents still found the results useful as they were perceived to either rule out the possibility of food allergy, provide reassurance they were currently acting correctly, or confirm what they already suspected. This gave them a feeling of control over the condition.

"It confirmed what I thought in a way I would have been surprised if she was allergic to something." (Parent 9)

"It was beneficial, so I know now if and when she ever starts eating eggs… we know she's fine with it." (Parent 3)

"It makes a difference. It doesn't change how I treat it but we had a negative result…but it definitely made a difference in terms of ok put your mind to rest…made us a bit more relaxed…And also maybe more feeling of being in control." (Parent 14)

However, some parents had mixed feelings about a negative test result: they were still in the position of uncertainty. They still did not know what causes the eczema or how to manage it, and while some parents were pleased that the test was negative, others were disappointed not to have any answers.

"So, it was mixed emotions, it was like ok that's good but still don't know what's causing it." (Parent 9)

For peer review only - http://bmjopen.bmj.com/site/about/guidelines.xhtml

"If it turned out he did have a dairy allergy, we would know that his diet definitely needed to be adapted, whereas I was just guessing most of the time…but then essentially the result of the allergy test was that he wasn't allergic to anything and I was still in the same situation at that point." (Parent 16)

Some parents appeared to accept that their child was "not allergic" to the foods tested but still had doubts about other foods not tested for.

"I still don't know if he has an allergy to anything that wasn't tested, 'cos they only test for certain ones." (Parent 16)

One parent whose child had received a negative allergy test result still felt that food was a factor. They believed the eczema to be an intolerance to food rather than an allergy, and so the allergy test would not have captured this.

"He didn't have an allergic reaction to milk, they didn't test him on soya…I never thought he was allergic to it, I just assumed he had an intolerance." (Parent 8)

DISCUSSION

Summary

We found different uncertainties among parents and GPs regarding the value of food allergy testing in children with eczema. Parents' beliefs around the causes of eczema, including the role of food allergy, and their information sources on this were mixed. Parents expressed few concerns about the limitations of allergy testing, and most were satisfied with the results which gave them a sense of control over their child's condition. Test results gave them confidence to not change their child's diet but sometimes left them with a desire for more information. GPs felt reluctant to refer for allergy testing due to uncertainty about the effect of testing and dietary management on eczema symptoms.

Strengths and limitations

As far as we are aware, this is the first qualitative study to specifically explore the views of parents and GPs regarding the role of food allergy in childhood eczema. We interviewed GPs and parents with a range of characteristics and employed a topic guide flexibly to help ensure that all aspects of the role of food allergy and testing in children with eczema were captured. However, all participants were either taking part in (parents) or hosting (GPs) the trial, meaning that to some extent they were

For peer review only - http://bmjopen.bmj.com/site/about/guidelines.xhtml

For peer review only

all open to the idea of children with eczema undergoing SPTs. We did not interview GPs from surgeries or parents who declined to take part in the trial. We only interviewed GPs and other healthcare professionals may have different experiences and views. In addition, a high proportion of parent participants were educated to degree level or higher (~60% in the trial); and only one parent interviewed had received a "positive" test result.

Comparison with existing literature

Evidence related to parents' food allergy knowledge, attitudes and beliefs is limited, but we know that parents are frustrated by inconsistent or contradictory messages from different doctors, (20-22) and that information online about diet and eczema is readily accessible but often inaccurate or misleading. (22) Our findings are consistent with a recent qualitative synthesis of the eczema literature, which identified a diverse range of beliefs about underlying causes and found that parents sought dietary avoidance as a potential "cure", removing the need for long-term treatment. (Teasdale E, Muller I, Sivyer K, Ghio D, Greenwell K, Wilczynska S, et al. Views and experiences of managing eczema: systematic review and thematic synthesis of qualitative studies. British Journal of Dermatology 2020) Parents in this study were motivated to have food allergy testing to help identify a cure and to ensure they were acting appropriately by including or excluding certain foods. We have reported previously that GPs often either avoid the topic of food allergy in eczema or, if raised, dissuade parents away from testing. (5, 23) Our study indicates this may be due to a lack of experience and understanding of food allergy testing. As per Halls et al's. (11) analysis of online forums, we identified parents' concerns that dietary restrictions may result in nutritional deficiencies or promote picky eating habits. Our findings suggest the perceived benefits of food allergy testing generally outweigh concerns and lead to parents engaging with food allergy testing.

Implications for research and practice

Our findings support the need for a definitive trial of test-guided dietary management for childhood eczema. Good quality evidence and resources are needed to guide GPs on how to advise parents regarding food allergy testing. There needs to be better quantification of where and how commonly parents seek dietary advice for eczema, what changes they make and what the implications for their child and family may be. The views and experiences of a wider range of healthcare professionals also needs to be captured.

Meanwhile, until better evidence emerges GPs should continue to follow guidance (2, 8) on food allergy testing in children with eczema, specifically seeking specialist advice where it is suspected

For peer review only - http://bmjopen.bmj.com/site/about/guidelines.xhtml

clinically because of immediate reactions, where there are suggestive symptoms in other organ systems or where the disease is difficult to treat despite optimal topical therapy. Parents are likely to benefit from signposting towards high quality evidence-based information regardless of whether allergy testing is indicated, to help them understand and manage their child's eczema. Alterations to children's diets should be done in conjunction with appropriately trained health care professional's advice to avoid unnecessary restrictions.

FUNDING

This study was funded by National Institute for Health Research (NIHR) School for Primary Care Research (project 383). MJR was funded by an NIHR Post-Doctoral Research Fellowship (PDF-2014-07-013). This study was designed and delivered in collaboration with the Bristol Randomised Trials Collaboration (BRTC), a UKCRC registered clinical trials unit which, as part of the Bristol Trials Centre, is in receipt of National Institute for Health Research CTU support funding. The views expressed in this article are those of the authors and not necessarily those of the NHS, NIHR, or the Department of Health and Social Care.

ETHICAL APPROVAL

The study has been reviewed by the Health Research Authority and given a favourable opinion by the NHS REC (West Midland-South Birmingham Ethics Committee, Reference Number 18/WM/0124)

COMPETING INTERESTS

MJR: No financial interests; convenes the NIHR SPCR Allergy working group; and was a member of the NICE Quality Standard 44 for Atopic eczema in under 12s and RCPCH "Care pathway for children with eczema" groups.

RJB: RJB has received honoraria for participating in advisory boards for ALK-Abello who manufacture allergy diagnostics and treatments, and DBV technologies and Prota therapeutics who develop food allergy treatments. RJB has undertaken expert witness work in legal cases concerning food anaphylaxis or infant formula health claims.

For peer review only - http://bmjopen.bmj.com/site/about/guidelines.xhtml

ACKNOWLEDGEMENTS

We would like to thank all the GPs and parent that participated in study; West of England Clinical Research Network; and to Catherine Gray, Jo McMeechan and the TEST/BEE Studies PPI group for their contributions. The study was developed with support from UK Dermatology Clinical Trials Network (UK DCTN). The UK DCTN is grateful to the British Association of Dermatologists and the University of Nottingham for financial support of the Network.

CONTRIBUTIONS

MJR conceived the study idea in collaboration with RJB, MS and IM; MJR, RJB, MS, IM, ARGS and KR developed the initial study design with later input from LS and CC on the nested qualitative study. CC and KR conducted the interviews. CC analysed the data with input from ARGS, LS, MJR and KR. CC prepared the manuscript, MJR, KR, MS, RJB, IM, AG, EA, LS and ARGS contributed to drafts of the paper and approved the final draft. CC finalised the paper for submission to the journal. All authors read and approved the final manuscript.

REFERENCES

1. Eichenfield LF, Tom WL, Chamlin SL, Feldman SR, Hanifin JM, Simpson EL, et al. Guidelines of care for the management of atopic dermatitis: section 1. Diagnosis and assessment of atopic dermatitis. J Am Acad Dermatol. 2014;70(2):338-51.
2. NICE. Atopic eczema in under 12s: diagnosis and management. London: National Institute for Health and Clinical Excellence; 2007.
3. Santer M, Burgess H, Yardley L, Ersser S, Lewis-Jones S, Muller I, et al. Experiences of carers managing childhood eczema and their views on its treatment: a qualitative study. British Journal of General Practice. 2012;62(597):e261.
4. Santer M, Burgess H, Yardley L, Ersser SJ, Lewis-Jones S, Muller I, et al. Managing childhood eczema: qualitative study exploring carers' experiences of barriers and facilitators to treatment adherence. J Adv Nurs. 2013;69(11):2493-501.
5. Powell K, Le Roux E, Banks JP, Ridd MJ. Developing a written action plan for children with eczema: a qualitative study. British Journal of General Practice. 2018;68(667):e81.
6. Beattie PE, Lewis-Jones MS. Parental knowledge of topical therapies in the treatment of childhood atopic dermatitis. Clinical and Experimental Dermatology. 2003;28(5):549-53.
7. Chan J, Ridd MJ. Beliefs and practices among adults with eczema and carers of children with eczema regarding the role of food allergy. Clin Exp Dermatol. 2019;44(7):e235-e7.
8. NICE. Food allergy in under 19s: assessment and diagnosis. London: National Institute for Health and Care Excellence; 2011.
9. Luyt D, Ball H, Kirk K, Stiefel G. Diagnosis and management of food allergy in children. Paediatrics and Child Health. 2016;26(7):287-91.
10. Lever R, MacDonald C, Waugh P, Aitchison T. Randomised controlled trial of advice on an egg exclusion diet in young children with atopic eczema and sensitivity to eggs. Pediatric Allergy and Immunology. 1998;9(1):13-9.

For peer review only - http://bmjopen.bmj.com/site/about/guidelines.xhtml

11. Halls A, Nunes D, Muller I, Angier E, Grimshaw K, Santer M. 'Hope you find your 'eureka' moment soon': a qualitative study of parents/carers' online discussions around allergy, allergy tests and eczema. BMJ Open. 2018;8(11):e022861.
12. Ridd MJ, Edwards L, Santer M, Chalmers JR, Waddell L, Marriage D, et al. TEST (Trial of Eczema allergy Screening Tests): protocol for feasibility randomised controlled trial of allergy tests in children with eczema, including economic scoping and nested qualitative study. BMJ Open. 2019;9(5):e028428.
13. Charman CR, Venn AJ, Ravenscroft JC, Williams HC. Translating Patient-Oriented Eczema Measure (POEM) scores into clinical practice by suggesting severity strata derived using anchor-based methods. Br J Dermatol. 2013;169(6):1326-32.
14. Department of Communities and Local Government. The English Index of Multiple Deprivation (IMD) 2015 — guidance 20152015 6th March, 2020. Available from: https://assets.publishing.service.gov.uk/government/uploads/system/uploads/attachment_data/file/464430/English_Index_of_Multiple_Deprivation_2015_-_Guidance.pdf.
15. Malterud K, Siersma VD, Guassora AD. Sample Size in Qualitative Interview Studies: Guided by Information Power. Qual Health Res. 2016;26(13):1753-60.
16. QSR International. What is NVivo? | NVivo 2019 [Available from: https://www.qsrinternational.com/nvivo-qualitative-data-analysis-software/about/nvivo.
17. Braun V, Clarke V. Using thematic analysis in psychology. Qualitative Research in Psychology. 2006;3(2):77-101.
18. Tracy SJ. Qualitative Quality: Eight “Big-Tent” Criteria for Excellent Qualitative Research. Qualitative Inquiry. 2010;16(10):837-51.
19. Leventhal H, Phillips LA, Burns E. The Common-Sense Model of Self-Regulation (CSM): a dynamic framework for understanding illness self-management. J Behav Med. 2016;39(6):935-46.
20. Goossens NJ, Flokstra-de Blok BM, van der Meulen GN, Botjes E, Burgerhof HG, Gupta RS, et al. Food allergy knowledge of parents - is ignorance bliss? Pediatr Allergy Immunol. 2013;24(6):567-73.
21. Gupta RS, Springston EE, Smith B, Kim JS, Pongracic JA, Wang X, et al. Food allergy knowledge, attitudes, and beliefs of parents with food-allergic children in the United States. Pediatr Allergy Immunol. 2010;21(6):927-34.
22. Khanna R, Shifrin N, Nektalova T, Goldenberg G. Diet and dermatology: Google search results for acne, psoriasis, and eczema. Cutis. 2018;102(1):44;6;8.
23. Le Roux E, Powell K, Banks JP, Ridd MJ. GPs’ experiences of diagnosing and managing childhood eczema: a qualitative study in primary care. British Journal of General Practice. 2018;68(667):e73.

For peer review only

For peer review only - http://bmjopen.bmj.com/site/about/guidelines.xhtml

BOX AND TABLES

Box 1 Topics explored in Parent and GP interviews

Parent Interview	GP interviews
• Beliefs about food allergy and their origin<br>• Perceived or experienced acceptability of allergy investigations, including skin prick tests<br>• Facilitators of and barriers to uptake of skin prick tests and dietary advice<br>• Worry or social difficulties related to food allergies<br>• Strategies used to manage their child's eczema, e.g. excluding foods	• Beliefs about food allergy testing<br>• Views of the acceptability of allergy tests to parents<br>• Facilitators of and barriers to uptake of allergy investigations (including blood and skin prick tests) and dietary advice in primary care

For peer review only

For peer review only - http://bmjopen.bmj.com/site/about/guidelines.xhtml

Table 1 Parent participant characteristics (n=21)

Parent Characteristics	Number of participants
**Trial arm allocation**	
Intervention	15
Comparator	6
**Child's POEM score**	
Mild/moderate (<17)	16
Severe (>17)	5
**Area deprivation score***	
Low	5
Medium	8
High	8
**SPT results**	
Negative	14
Positive	1
N/A Comparator	6
**Education Level**	
Degree or higher	13
Diploma	1
A-level	3
G.C.S.E	2
NVQ	2

***Index of Multiple Deprivation (14) based on home postcode**

Table 2 GP participant characteristics (n=11)

GP Characteristics	Number of participants
**Years' experience**	
0-5	3
6-10	3
11-15	4
16-21	0
21+	1
**Confidence in managing eczema***	
Low	0
Medium	5
High	6
**Practice deprivation score****	6
Low	1
Medium	4
High	

***Self-reported scale 1-10, low=1-3, medium=4-7, high=8-10**

****Index of Multiple Deprivation (14)**

For peer review only - http://bmjopen.bmj.com/site/about/guidelines.xhtml

COREQ (COnsolidated criteria for REporting Qualitative research) Checklist

A checklist of items that should be included in reports of qualitative research. You must report the page number in your manuscript where you consider each of the items listed in this checklist. If you have not included this information, either revise your manuscript accordingly before submitting or note N/A.

Topic	Item No.	Guide Questions/Description	Reported on Page No.
**Domain 1: Research team and reflexivity**			
Personal characteristics			
Interviewer/facilitator	1	Which author/s conducted the interview or focus group?	5
Credentials	2	What were the researcher's credentials? E.g. PhD, MD	1
Occupation	3	What was their occupation at the time of the study?	5
Gender	4	Was the researcher male or female?	n/a
Experience and training	5	What experience or training did the researcher have?	5
Relationship with participants			
Relationship established	6	Was a relationship established prior to study commencement?	
Participant knowledge of the interviewer	7	What did the participants know about the researcher? e.g. personal goals, reasons for doing the research	
Interviewer characteristics	8	What characteristics were reported about the inter viewer/facilitator? e.g. Bias, assumptions, reasons and interests in the research topic	
**Domain 2: Study design**			
Theoretical framework			
Methodological orientation and Theory	9	What methodological orientation was stated to underpin the study? e.g. grounded theory, discourse analysis, ethnography, phenomenology, content analysis	
Participant selection			
Sampling	10	How were participants selected? e.g. purposive, convenience, consecutive, snowball	5
Method of approach	11	How were participants approached? e.g. face-to-face, telephone, mail, email	5
Sample size	12	How many participants were in the study?	6
Non-participation	13	How many people refused to participate or dropped out? Reasons?	
Setting			
Setting of data collection	14	Where was the data collected? e.g. home, clinic, workplace	5
Presence of non-participants	15	Was anyone else present besides the participants and researchers?	
Description of sample	16	What are the important characteristics of the sample? e.g. demographic data, date	6
Data collection			
Interview guide	17	Were questions, prompts, guides provided by the authors? Was it pilot tested?	5
Repeat interviews	18	Were repeat inter views carried out? If yes, how many?	
Audio/visual recording	19	Did the research use audio or visual recording to collect the data?	6
Field notes	20	Were field notes made during and/or after the inter view or focus group?	
Duration	21	What was the duration of the inter views or focus group?	
Data saturation	22	Was data saturation discussed?	5
Transcripts returned	23	Were transcripts returned to participants for comment and/or	

For peer review only

For peer review only - http://bmjopen.bmj.com/site/about/guidelines.xhtml

Topic	Item No.	Guide Questions/Description	Reported on Page No.
		correction?	
**Domain 3: analysis and findings**			
Data analysis			
Number of data coders	24	How many data coders coded the data?	6
Description of the coding tree	25	Did authors provide a description of the coding tree?	
Derivation of themes	26	Were themes identified in advance or derived from the data?	6
Software	27	What software, if applicable, was used to manage the data?	6
Participant checking	28	Did participants provide feedback on the findings?	
Reporting			
Quotations presented	29	Were participant quotations presented to illustrate the themes/findings? Was each quotation identified? e.g. participant number	6-13
Data and findings consistent	30	Was there consistency between the data presented and the findings?	6-14
Clarity of major themes	31	Were major themes clearly presented in the findings?	6-13
Clarity of minor themes	32	Is there a description of diverse cases or discussion of minor themes?	

Developed from: Tong A, Sainsbury P, Craig J. Consolidated criteria for reporting qualitative research (COREQ): a 32-item checklist for interviews and focus groups. *International Journal for Quality in Health Care*. 2007. Volume 19, Number 6: pp. 349 – 357

Once you have completed this checklist, please save a copy and upload it as part of your submission. DO NOT include this checklist as part of the main manuscript document. It must be uploaded as a separate file.

For peer review only

BMJ Open

Parents and GPs' understandings and beliefs about food allergy testing in children with eczema: qualitative interview study within the Trial of Eczema allergy Screening Tests (TEST) feasibility trial

Journal:	*BMJ Open*
Manuscript ID	bmjopen-2020-041229.R1
Article Type:	Original research
Date Submitted by the Author:	11-Aug-2020
Complete List of Authors:	Clement, Clare; University of Bristol, Bristol Medical School<br>Ridd, Matthew; University of Bristol Faculty of Health Sciences, Population Health Sciences;<br>Roberts, Kirsty; University of Bristol School of Social and Community Medicine<br>Santer, Miriam; University of Southampton, Primary Care & Population Sciences<br>Boyle, R; Imperial College London; University of Nottingham<br>Muller, Ingrid; University of Southampton, Department of Psychology<br>Gilbertson, Anna; University of Bristol, School Of Social & Community Medicine<br>Angier, Elizabeth; University of Southampton, Primary Care & Population Sciences<br>Selman, Lucy; University of Bristol School of Social and Community Medicine<br>Shaw, Ali; University of Bristol Faculty of Arts, Bristol Medical School
**Primary Subject Heading**:	Dermatology
Secondary Subject Heading:	General practice / Family practice, Immunology (including allergy), Paediatrics
Keywords:	Eczema < DERMATOLOGY, NUTRITION & DIETETICS, PRIMARY CARE, Allergy < THORACIC MEDICINE, QUALITATIVE RESEARCH, Community child health < PAEDIATRICS

SCHOLARONE™
Manuscripts

I, the Submitting Author has the right to grant and does grant on behalf of all authors of the Work (as defined in the below author licence), an exclusive licence and/or a non-exclusive licence for contributions from authors who are: i) UK Crown employees; ii) where BMJ has agreed a CC-BY licence shall apply, and/or iii) in accordance with the terms applicable for US Federal Government officers or employees acting as part of their official duties; on a worldwide, perpetual, irrevocable, royalty-free basis to BMJ Publishing Group Ltd ("BMJ") its licensees and where the relevant Journal is co-owned by BMJ to the co-owners of the Journal, to publish the Work in this journal and any other BMJ products and to exploit all rights, as set out in our licence.

The Submitting Author accepts and understands that any supply made under these terms is made by BMJ to the Submitting Author unless you are acting as an employee on behalf of your employer or a postgraduate student of an affiliated institution which is paying any applicable article publishing charge ("APC") for Open Access articles. Where the Submitting Author wishes to make the Work available on an Open Access basis (and intends to pay the relevant APC), the terms of reuse of such Open Access shall be governed by a Creative Commons licence – details of these licences and which Creative Commons licence will apply to this Work are set out in our licence referred to above.

Other than as permitted in any relevant BMJ Author's Self Archiving Policies, I confirm this Work has not been accepted for publication elsewhere, is not being considered for publication elsewhere and does not duplicate material already published. I confirm all authors consent to publication of this Work and authorise the granting of this licence.

For peer review only

Parents and GPs' understandings and beliefs about food allergy testing in children with eczema: qualitative interview study within the Trial of Eczema allergy Screening Tests (TEST) feasibility trial

Authors:

Clare Clement MSc[1], Matthew J Ridd PhD[2], Kirsty Roberts PhD[2], Miriam Santer PhD[3], Robert J Boyle PhD[4, 5], Ingrid Muller PhD[3], Anna Gilbertson MSc[2], Elizabeth Angier MSc[3], Lucy Selman PhD[1,2], Alison R G Shaw PhD[2]

[1] Bristol Trials Centre, Bristol Medical School, University of Bristol, Bristol, UK

[2] Population Health Sciences, University of Bristol, UK

[3] Primary Care, Population Sciences & Medical Education, University of Southampton, Southampton, UK

[4] Imperial College London, London, UK

[5] Centre of Evidence-Based Dermatology, University of Nottingham, Nottingham, UK

Corresponding author:

Clare Clement, Bristol Trials Centre, Bristol Medical School, University of Bristol, Bristol, UK.
c.clement@bristol.ac.uk, (0117) 9287269

Keywords

Eczema, parents, diet, child, primary care practice, allergens, qualitative

Word Count: 4044

For peer review only - http://bmjopen.bmj.com/site/about/guidelines.xhtml

ABSTRACT

Aim: To explore parent and General Practitioner (GP) understanding and beliefs about food allergy testing for children with eczema.

Design and Setting: Qualitative interview study in UK primary care within the Trial of Eczema allergy Screening Tests (TEST) feasibility trial.

Participants: Semi-structured interviews with parents of children with eczema taking part in the feasibility study and GPs at practices hosting the study.

Results: 21 parents and 11 GPs were interviewed. Parents discussed a range of potential causes for eczema, including a role for food allergy. They believed allergy testing to be beneficial as it could potentially identify a cure or help reduce symptoms and they found negative tests reassuring, suggesting to them that no dietary changes were needed. GPs reported limited experience and uncertainty regarding food allergy in children with eczema. While some GPs believed referral for allergy testing could be appropriate, most were unclear about its utility. They thought it should be reserved for children with severe eczema or complex problems but wanted more information to advise parents and help guide decision-making.

Conclusions: Parents' motivations for allergy testing are driven by the desire to improve their child's condition and exclude food allergy as a possible cause of symptoms. GPs are uncertain about the role of allergy testing and want more information about its usefulness to support parents and help inform decision making.

Trial registration: ISRCTN15397185

For peer review only - http://bmjopen.bmj.com/site/about/guidelines.xhtml

STRENGTHS AND LIMITATIONS OF THIS STUDY

- We believe this is the first qualitative study to specifically explore GPs' and parents' views regarding the role of food allergy in children with eczema.
- We interviewed GPs and parents with a range of characteristics and employed a topic guide flexibly to ensure that different aspects of food allergy and testing in children with eczema were captured.
- By virtue of taking part in the trial, to some extent all participants were open to the idea of children with eczema undergoing Skin Prick Tests for food allergies.
- Other healthcare professionals and less well-educated parents may have different experiences and opinions.

For peer review only

For peer review only - http://bmjopen.bmj.com/site/about/guidelines.xhtml

INTRODUCTION

Eczema (synonyms atopic eczema/dermatitis) is a common and burdensome condition, especially among pre-school age children. (1) Clinical guidelines emphasise the importance of avoiding environmental irritants and practising good skin care through regular use of emollients and appropriate use of topical corticosteroids. (2)

A concern among parents of children with eczema, voiced to GPs and commonly seen in online forums, is the role of food allergy. (3-5) Despite weak evidence to support dietary modification, many parents try excluding foods from their child's diet to reduce eczema symptoms or the need for treatment with medications. (6, 7)

In the UK, NICE guidance (2, 8) recommends that healthcare professionals consider food allergies as potential triggers in children with eczema if they develop symptoms immediately after ingesting a potential allergen, or in those with moderate to severe eczema who have not responded to optimum management. Immediate-type food allergies are more common in children with eczema (9, 10) and food allergy testing may potentially prevent serious allergic reactions and/or identify foods causing eczema symptoms. However, in primary care professional advice as to the importance of allergy testing for children with eczema is variable, as is access to allergy testing. (11) In principle, if allergy testing were shown to inform eczema care then it could be routinely offered in primary care. However, the effectiveness of food allergy testing and associated dietary measures for managing eczema is uncertain.

In addition, there is also uncertainty about the feasibility and acceptability of conducting research to answer the question of if, or when, food allergy testing should be routinely offered to children with eczema. It is important to resolve these uncertainties to help inform parents and GPs decision making about optimising diet and management of eczema.

Nested within the feasibility Trial of Eczema allergy Screening Tests Study (TEST) study, (12) we report on a qualitative study that explored parent and GP understanding and beliefs about food allergy testing for children with eczema.

For peer review only - http://bmjopen.bmj.com/site/about/guidelines.xhtml

METHOD

Study design

The TEST study was conducted to determine the feasibility of conducting a trial comparing test-guided dietary management versus usual care, for the management of eczema in children. More detail can be found elsewhere, (12) but in brief it was a single centre, two-group, individually randomised, feasibility randomised controlled trial conducted in 17 GP surgeries in the West of England. Children aged between >3 months and <5 years with mild or worse eczema were randomised to either control (usual care) or intervention. The intervention comprised a structured allergy history and Skin Prick Tests (SPTs) for cow's milk, hen's egg, wheat, peanut, cashew, and codfish. Dietary advice was given, based on test results, to continue eating/introduce as normal or to try excluding and reintroducing one or more foods from the child's diet. Where appropriate, referral was made for an oral food challenge, where the child was exposed to a potential allergen under supervision, (12) or to a local allergy clinical for review. All participants were followed-up for six months.

Semi-structured interviews were conducted with a sample of parents of children in the TEST trial and GPs from participating practices. The aim was to explore participants' beliefs about the role of food allergy in children with eczema, the acceptability of testing and potential barriers to and facilitators of the uptake of food allergy testing in primary care.

Sampling and recruitment

Purposive sampling was used to capture maximum variation in views and experiences. Parents were sampled from both the intervention and usual care groups and reflected mild/moderate (<17) vs severe (≥17) Patient Orientated Eczema Measure (POEM) symptom score, (13) and socio-economic status (assessed using the Index of Multiple Deprivation Decile (IMDD) (categories: high (8-10)/ medium (5-7)/low (1-4)) (14). Participants allocated to the intervention group were also sampled based on whether they had a negative or positive SPT results. The sampling of GPs captured variation in IMDD (14) of the practice postcode and doctor characteristics (length of time practicing as a GP and self-reported confidence in managing eczema (scale 1-10, 1=low, 10=high). Sample size was informed by the team's judgement that we had enough "information power" to meet the study aims. (15)

For peer review only - http://bmjopen.bmj.com/site/about/guidelines.xhtml

Data collection

Interviews were conducted face-to-face and via telephone by CC (17 parents, 11 GPs), an experienced social science researcher and KR (4 parents), the Trial Manager with experience of qualitative research. Written consent was obtained for the face-to-face interviews and verbal consent was recorded for the telephone interviews. Interviews lasted between 16 and 43 minutes (mean 25 minutes). No notable differences in length or depth of data were seen between face-to-face (6 parents) and telephone interviews (15 parents. 11 GPs). A flexible, semi-structured topic guide was used to assist questioning but allow participants to introduce and discuss new issues (Box 1). The full topic guide is available as supplementary material.

Data analysis

Interviews were audio-recorded, transcribed verbatim, anonymised, and imported to Nvivo10 (16) for data management and coding. Analysis started shortly after data collection started and analytical insights were fed back into further data collection and analysis. Transcripts were analysed thematically using both inductive and deductive coding. (17) Transcripts were coded to establish an initial coding framework and study team members (CC, ARGS, KR and MJR) each independently coded a sub-set of seven transcripts; any discrepancies were discussed to ensure a coding consensus and maximise rigour. (18) The framework was then applied to all the remaining transcripts by CC. Emergent findings were discussed in regular multi-disciplinary Trial Management Group meetings to enhance validity. After coding was completed we drew on the Common-Sense Model to help interpret findings. (19)

Patient and Public Involvement (PPI)

Two parents of children with eczema were members of the Trial Management Group and advised on qualitative data collection and analysis. In addition, PPI feedback was the incorporated into the final topic guide.

RESULTS

Twenty-one parents were interviewed from eleven trial practices (Table 1). Eleven GPs were interviewed from seven trial practices (Table 2). Four main themes emerged from the analysis (two of which related to parent data only): *Parents' causes and associations, Knowledge and awareness, Searching for a 'cure' or seeking reassurance around current dietary practice,* and *Parents' responses to food allergy test results*.

For peer review only - http://bmjopen.bmj.com/site/about/guidelines.xhtml

Parents' causes and associations

Parents seemed unsure of the causes of eczema and discussed several possible factors. They received advice and information from a wide range of sources such as other parents, doctors, family members and the media. Some parents acted on this information, including by removing foods from their child's diet.

Parents believed that family history, age or environmental factors were responsible for their child's eczema, with some discussing how factors could interact or vary depending on the individual.

"I think it must be like a heat thing…it was just to see if something that is genetic that my wife had when she was little and then she grew out of it…But that's really everything we know… But I do have a lot of allergies, so I don't know if she's inherited it." (Parent 1)

"To be honest I don't know an awful lot…I think that obviously dry skin, like water does affect it, but what actually causes it I don't know whether its stuff in the environment…I think there are different causes for different people as well." (Parent 7)

Parents reported being influenced by media coverage of eczema and anecdotal stories from other parents whose child's symptoms improved when certain foods, particularly dairy, were eliminated from their diet.

"I've got a friend that her little girl…was actually allergic to dairy so that resolved some of her skin problems…and so being told by a friend actually dairy's not good for them and so you prevent them eating certain things…and that will resolve the problem." (Parent 9)

Some parents had been alerted to the possible role of food allergies by speaking with health care professionals, including GPs and health visitors, about eczema, who had suggested trying elimination diets or keeping a food diary.

"That's what the doctor told us anyway, they said we might need to consider food allergies…they did say that if babies already have allergies, say like a milk allergy, then certain things can be a problem…I think they mentioned soya as well…they said a food diary would be a good idea." (Parent 2)

For peer review only - http://bmjopen.bmj.com/site/about/guidelines.xhtml

"The health visitor proposed, she suggested that, to run an elimination diet." (Parent 21)

Several parents had explored the role of food in their own child's eczema, before taking part in TEST. In most parent accounts this did not improve the child's condition or improvement was not clearly linked to the change in diet.

"It may be food…so we did quite a lot of changing things at the time…we tried keeping a food diary…I think we started off offering a new thing every couple of days just to see if she reacted to it…there was nothing which obviously made her significantly worse….we thought maybe eggs but then we reintroduced them…and she was fine." (Parent 2)

"Whether it was a natural improvement, or it was the milk-free diet, I can't say but that's what happened at that point." (Parent 21)

Some parents, although aware of such stories, were not convinced and wanted more 'evidence' before they acted.

"Probably someone has said to me have you tried cutting out dairy and I haven't…If I actually listen to people with their anecdotal things or…my friend's child had eczema and they stopped them having dairy, but I'm a little bit cynical." (Parent 7)

"Everyone always says oh there's a link between dairy and eczema, but we could never really find any studies or like have it proven…there's a lot of hearsay but it hasn't actually been proven." (Parent 10)

Some parents believed food allergy may be a factor in their child's eczema by observing a link between their child eating certain foods and their eczema getting worse or eczema occurring when foods were introduced. Others did not see a role or reported having independently "ruled it out" through exclusion of certain foods from their child's diet.

"I used to find if she drank certain things…she would start to scratch and then her eczema started to flare up. Sometimes I would notice after she'd eaten chocolate or something she'd flare up as well." (Parent 3)

"I did strongly think it was to do with foods because of when I stopped breastfeeding it was something that flared up." (Parent 12)

For peer review only - http://bmjopen.bmj.com/site/about/guidelines.xhtml

"I tried cow's milk, all fish, banana…and nothing, no changes…It's not food. I'm sure it's not food." (Parent 17)

Knowledge and awareness

Knowledge and awareness of food allergy and food allergy testing and its role in managing eczema varied across both parents and GPs. Some parents were aware of what food allergies were before the trial and named common allergens, with some reporting personal experience of such food allergies. However, some parents still reported limited knowledge and understanding of food allergies.

"I know there's loads of them. The main ones are dairy and gluten and nuts. That's probably it as far as my knowledge goes." (Parent 17)

"I'm not really clued up on much of it…I have certain foods myself my tongue flares up…but other than that I don't really have any clue of food allergies." (Parent 12)

Parents and GPs labelled a food allergy as a set of acute or severe symptoms which could arise from a potential reaction to food. Symptoms which were perceived to be less severe, delayed or gastrointestinal upset were labelled as an intolerance rather than allergy.

"So I would say true allergy causes an allergic reaction, so causes an ideated reaction, so usually presents as sort of problems with breathing, lip swelling, skin response so like hives, urticaria, whereas I think intolerance seems to come with GI upset or sometimes can have skin reactions but tends not to create a full blown allergic response." (GP 9)

"I think an intolerance [is] just where it might upset them a little bit, you might get mild stomach cramps… whereas an allergy where they mouth might swell up or affect their breathing…more severe." (Parent 11)

Parents who had asked about allergy testing reported frustration with responses from GPs who believed the child did not have severe enough eczema to warrant testing.

"I asked if he could have allergy testing just to make sure it wasn't anything like that, but they said they don't tend to do it in young children unless it's [eczema] like severe." (Parent 8)

For peer review only - http://bmjopen.bmj.com/site/about/guidelines.xhtml

One parent commented on how they felt that GPs did not have the appropriate information to advise parents:

> *"I don't think there's any solid research to say whether or not there is a link so…it's difficult for doctors to advise on something there isn't any evidence for." (Parent 8)*

GPs reported limited experience of and knowledge about food allergy in general and wanted more information to guide their decision making for allergy testing in children with eczema.

> *"[A] minefield is how I would put it, I think. It's not something we're taught well at medical school…there's a massive online presence about allergy testing, much of it not evidence based. So, there's a big unmet need with parents coming with questions and I think doctors have an unmet educational need." (GP 5)*

GPs reported parents frequently requesting food allergy tests for their children. Some more inexperienced GPs reported being uncertain about the evidence and found advising parents difficult. Few of the GPs had referred a child with eczema specifically for food allergy tests, preferring to refer them to a dermatologist for more general advice or telling parents to keep a food diary.

> *"I don't feel completely confident in knowing its [allergy testing] limitations and uses…we get parents requesting allergy testing and it sort of feels like my training has always suggest there isn't necessarily a role in most cases, but there might be a role in some cases, so I'm not completely clear about the evidence behind it so I find it a bit tricky to advise parents on that." (GP 3)*

Search for a 'cure' or seeking reassurance around current dietary practice

Parents' motivation and willingness for their child to have food allergy testing was influenced by a range of factors. While parents were uncertain of the role of food allergy in eczema, they discussed how food allergy testing might be beneficial in identifying a cause for their child's eczema, providing a cure or helping with management of the condition.

> *"I just want to get on top of it [eczema] but you just can't because you don't know what is triggering it off…just to rule it [food allergy] out…then I can just stop him from having that food and then it won't bother him" (Parent 5)*

For peer review only - http://bmjopen.bmj.com/site/about/guidelines.xhtml

For peer review only

They also appeared to seek reassurance or support for their current dietary management strategy; for example, they wanted to know they were doing "the right thing" for their child by excluding or not excluding foods:

> *"I'd really like to know if, because I've had so many people say to me about dairy and 'cos I haven't done it, so I'd like to know that I'm doing the right thing." (Parent 7)*

However, some parents did express concerns about the impact that identifying a food allergy could have on their family. There were concerns for balanced nutrition and difficulties accommodating different diets within families:

> *"You need to make sure that they're getting enough fats, particularly for children and protein and fibre and everything else and carbohydrates when you're excluding all of this, and it could be actually quite difficult for some parents, some parents might have other children so the other children are eating one thing and…have to do it for the whole household." (Parent 18)*

Some parents were concerned the skin prick test could be uncomfortable for their child and were relieved their child was in the usual care group and did not have it.

> *"I was quite glad then to be honest he didn't have to do allergy testing…once I saw the fact that he wasn't that happy about being probed and prodded anyway I thought yeah, probably better off not having to do it." (Parent 7)*

Some GPs said they believed allergy testing to be appropriate for some children with eczema as it could be useful for informing potential dietary alterations to help manage the eczema. But most GPs, particularly those with more experience, had reservations about the usefulness of testing and were cautious about making referrals to allergy clinics. GPs reported being more likely to refer children for food allergy testing in severe or complex cases and where the cause of the eczema was not clear, and said they were often guided by parental wishes:

> *"I tend to discourage it [allergy testing] if I'm honest…I think unless we're having problems getting a child's eczema and their symptoms under control…I think in a child who would be very severely affected I would because I think well we're not getting this under control, we need more information…if there was a family history of food intolerance, allergies, those sort of situations." (GP 6)*

For peer review only - http://bmjopen.bmj.com/site/about/guidelines.xhtml

Whether parents or GPs thought the child may grow out of the eczema also influenced decisions to consider food allergy testing.

"Most children grow out of it and most of the time it's quite mild I think that most of the time people just tend to treat it and not perhaps think about the allergy side of things." (GP 1)

Parents' responses to food allergy test results

Parents in the intervention group expressed a range of responses to the food allergy test results. Most parents had faith in the healthcare professionals and the test and therefore accepted the results as being accurate.

"I couldn't see any reason not to trust it…I'm pretty trusting in professionals." (Parent 16)

Most results were negative, but parents still found the results useful as they were perceived to either rule out the possibility of food allergy, provide reassurance they were currently acting correctly, or confirm what they already suspected. This gave them a feeling of control over the condition.

"It confirmed what I thought in a way I would have been surprised if she was allergic to something." (Parent 9)

"It was beneficial, so I know now if and when she ever starts eating eggs… we know she's fine with it." (Parent 3)

"It makes a difference. It doesn't change how I treat it but we had a negative result…but it definitely made a difference in terms of ok put your mind to rest…made us a bit more relaxed…And also maybe more feeling of being in control." (Parent 14)

However, some parents had mixed feelings about a negative test result: they were still in the position of uncertainty. They still did not know what causes the eczema or how to manage it, and while some parents were pleased that the test was negative, others were disappointed not to have any answers.

"So, it was mixed emotions, it was like ok that's good but still don't know what's causing it." (Parent 9)

For peer review only - http://bmjopen.bmj.com/site/about/guidelines.xhtml

> *"If it turned out he did have a dairy allergy, we would know that his diet definitely needed to be adapted, whereas I was just guessing most of the time…but then essentially the result of the allergy test was that he wasn't allergic to anything and I was still in the same situation at that point." (Parent 16)*

Some parents appeared to accept that their child was "not allergic" to the foods tested but still had doubts about other foods not tested for.

> *"I still don't know if he has an allergy to anything that wasn't tested, 'cos they only test for certain ones." (Parent 16)*

One parent whose child had received a negative allergy test result still felt that food was a factor. They believed the eczema to be an intolerance to food rather than an allergy, and so the allergy test would not have captured this.

> *"He didn't have an allergic reaction to milk, they didn't test him on soya…I never thought he was allergic to it, I just assumed he had an intolerance." (Parent 8)*

DISCUSSION

Summary

We found different uncertainties among parents and GPs regarding the value of food allergy testing in children with eczema. Parents' beliefs around the causes of eczema, including the role of food allergy, and their information sources on this were mixed. Parents expressed few concerns about the limitations of allergy testing, and most were satisfied with the results which gave them a sense of control over their child's condition. Test results gave them confidence to not change their child's diet but sometimes left them with a desire for more information. GPs felt reluctant to refer for allergy testing due to uncertainty about the effect of testing and dietary management on eczema symptoms.

Strengths and limitations

As far as we are aware, this is the first qualitative study to specifically explore the views of parents and GPs regarding the role of food allergy in childhood eczema. We interviewed GPs and parents with a range of characteristics and employed a topic guide flexibly to help ensure that all aspects of the role of food allergy and testing in children with eczema were captured. However, all participants were either taking part in (parents) or hosting (GPs) the trial, meaning that to some extent they were

For peer review only - http://bmjopen.bmj.com/site/about/guidelines.xhtml

For peer review only

all open to the idea of children with eczema undergoing SPTs. We did not explore how age of parents or ethnicity of parents or GPs may influence beliefs about food allergies and eczema and practice. We did not interview GPs from surgeries or parents who declined to take part in the trial. We only interviewed GPs and other healthcare professionals may have different experiences and views. In addition, a high proportion of parent participants were educated to degree level or higher (~60% in the trial); and only one parent interviewed had received a "positive" test result.

Comparison with existing literature

Evidence related to parents' food allergy knowledge, attitudes and beliefs is limited, but we know that parents are frustrated by inconsistent or contradictory messages from different doctors, (20-22) and that information online about diet and eczema is readily accessible but often inaccurate or misleading. (22) Our findings are consistent with a recent qualitative synthesis of the eczema literature, which identified a diverse range of beliefs about underlying causes and found that parents sought dietary avoidance as a potential "cure", removing the need for long-term treatment. (23)Parents in this study were motivated to have food allergy testing to help identify a cure and to ensure they were acting appropriately by including or excluding certain foods. We have reported previously that GPs often either avoid the topic of food allergy in eczema or, if raised, dissuade parents away from testing. (5, 24) Our study indicates this may be due to a lack of experience and understanding of food allergy testing. As per Halls et al's. (11) analysis of online forums, we identified parents' concerns that dietary restrictions may result in nutritional deficiencies or promote picky eating habits. Our findings suggest the perceived benefits of food allergy testing generally outweigh concerns and lead to parents engaging with food allergy testing.

Implications for research and practice

Our findings support the need for a definitive trial of test-guided dietary management for childhood eczema. Good quality evidence and resources are needed to guide GPs on how to advise parents regarding food allergy testing. There needs to be better quantification of where and how commonly parents seek dietary advice for eczema, what changes they make and what the implications for their child and family may be. The views and experiences of a wider range of healthcare professionals, such as paediatric dermatologists or general paediatricians, also needs to be captured.

Meanwhile, until better evidence emerges GPs should continue to follow guidance (2, 8) on food allergy testing in children with eczema, specifically seeking specialist advice where it is suspected clinically because of immediate reactions, where there are suggestive symptoms in other organ

For peer review only - http://bmjopen.bmj.com/site/about/guidelines.xhtml

systems or where the disease is difficult to treat despite optimal topical therapy. Parents are likely to benefit from signposting towards high quality evidence-based information (25) regardless of whether allergy testing is indicated, to help them understand and manage their child's eczema. Alterations to children's diets should be done in conjunction with appropriately trained health care professional's advice to avoid unnecessary restrictions.

FUNDING

This study was funded by National Institute for Health Research (NIHR) School for Primary Care Research (project 383). MJR was funded by an NIHR Post-Doctoral Research Fellowship (PDF-2014-07-013). This study was designed and delivered in collaboration with the Bristol Randomised Trials Collaboration (BRTC), a UKCRC registered clinical trials unit which, as part of the Bristol Trials Centre, is in receipt of National Institute for Health Research CTU support funding. The views expressed in this article are those of the authors and not necessarily those of the NHS, NIHR, or the Department of Health and Social Care.

ETHICAL APPROVAL

The study has been reviewed by the Health Research Authority and given a favourable opinion by the NHS REC (West Midland-South Birmingham Ethics Committee, Reference Number 18/WM/0124)

COMPETING INTERESTS

MJR: No financial interests; convenes the NIHR SPCR Allergy working group; and was a member of the NICE Quality Standard 44 for Atopic eczema in under 12s and RCPCH "Care pathway for children with eczema" groups.

RJB: RJB has received honoraria for participating in advisory boards for ALK-Abello who manufacture allergy diagnostics and treatments, and DBV technologies and Prota therapeutics who develop food allergy treatments. RJB has undertaken expert witness work in legal cases concerning food anaphylaxis or infant formula health claims.

For peer review only - http://bmjopen.bmj.com/site/about/guidelines.xhtml

For peer review only

ACKNOWLEDGEMENTS

We would like to thank all the GPs and parent that participated in study; West of England Clinical Research Network; and to Catherine Gray, Jo McMeechan and the TEST/BEE Studies PPI group for their contributions. The study was developed with support from UK Dermatology Clinical Trials Network (UK DCTN). The UK DCTN is grateful to the British Association of Dermatologists and the University of Nottingham for financial support of the Network.

CONTRIBUTIONS

MJR conceived the study idea in collaboration with RJB, MS and IM; MJR, RJB, MS, IM, ARGS and KR developed the initial study design with later input from LS and CC on the nested qualitative study. CC and KR conducted the interviews. CC analysed the data with input from ARGS, LS, MJR and KR. CC prepared the manuscript, MJR, KR, MS, RJB, IM, AG, EA, LS and ARGS contributed to drafts of the paper and approved the final draft. CC finalised the paper for submission to the journal. All authors read and approved the final manuscript.

For peer review only

DATA AVAILABILITY STATEMENT

All data relevant to the study are included in the article or uploaded as supplementary information.

REFERENCES

1. Eichenfield LF, Tom WL, Chamlin SL, Feldman SR, Hanifin JM, Simpson EL, et al. Guidelines of care for the management of atopic dermatitis: section 1. Diagnosis and assessment of atopic dermatitis. J Am Acad Dermatol. 2014;70(2):338-51.
2. NICE. Atopic eczema in under 12s: diagnosis and management. London: National Institute for Health and Clinical Excellence; 2007.
3. Santer M, Burgess H, Yardley L, Ersser S, Lewis-Jones S, Muller I, et al. Experiences of carers managing childhood eczema and their views on its treatment: a qualitative study. British Journal of General Practice. 2012;62(597):e261.
4. Santer M, Burgess H, Yardley L, Ersser SJ, Lewis-Jones S, Muller I, et al. Managing childhood eczema: qualitative study exploring carers' experiences of barriers and facilitators to treatment adherence. J Adv Nurs. 2013;69(11):2493-501.
5. Powell K, Le Roux E, Banks JP, Ridd MJ. Developing a written action plan for children with eczema: a qualitative study. British Journal of General Practice. 2018;68(667):e81.
6. Beattie PE, Lewis-Jones MS. Parental knowledge of topical therapies in the treatment of childhood atopic dermatitis. Clinical and Experimental Dermatology. 2003;28(5):549-53.
7. Chan J, Ridd MJ. Beliefs and practices among adults with eczema and carers of children with eczema regarding the role of food allergy. Clin Exp Dermatol. 2019;44(7):e235-e7.

For peer review only - http://bmjopen.bmj.com/site/about/guidelines.xhtml

8. NICE. Food allergy in under 19s: assessment and diagnosis. London: National Institute for Health and Care Excellence; 2011.
9. Luyt D, Ball H, Kirk K, Stiefel G. Diagnosis and management of food allergy in children. Paediatrics and Child Health. 2016;26(7):287-91.
10. Lever R, MacDonald C, Waugh P, Aitchison T. Randomised controlled trial of advice on an egg exclusion diet in young children with atopic eczema and sensitivity to eggs. Pediatric Allergy and Immunology. 1998;9(1):13-9.
11. Halls A, Nunes D, Muller I, Angier E, Grimshaw K, Santer M. 'Hope you find your 'eureka' moment soon': a qualitative study of parents/carers' online discussions around allergy, allergy tests and eczema. BMJ Open. 2018;8(11):e022861.
12. Ridd MJ, Edwards L, Santer M, Chalmers JR, Waddell L, Marriage D, et al. TEST (Trial of Eczema allergy Screening Tests): protocol for feasibility randomised controlled trial of allergy tests in children with eczema, including economic scoping and nested qualitative study. BMJ Open. 2019;9(5):e028428.
13. Charman CR, Venn AJ, Ravenscroft JC, Williams HC. Translating Patient-Oriented Eczema Measure (POEM) scores into clinical practice by suggesting severity strata derived using anchor-based methods. Br J Dermatol. 2013;169(6):1326-32.
14. Department of Communities and Local Government. The English Index of Multiple Deprivation (IMD) 2015 — guidance 20152015 6th March, 2020. Available from: https://assets.publishing.service.gov.uk/government/uploads/system/uploads/attachment_data/file/464430/English_Index_of_Multiple_Deprivation_2015_-_Guidance.pdf.
15. Malterud K, Siersma VD, Guassora AD. Sample Size in Qualitative Interview Studies: Guided by Information Power. Qual Health Res. 2016;26(13):1753-60.
16. QSR International. What is NVivo? | NVivo 2019 [Available from: https://www.qsrinternational.com/nvivo-qualitative-data-analysis-software/about/nvivo.
17. Braun V, Clarke V. Using thematic analysis in psychology. Qualitative Research in Psychology. 2006;3(2):77-101.
18. Tracy SJ. Qualitative Quality: Eight “Big-Tent” Criteria for Excellent Qualitative Research. Qualitative Inquiry. 2010;16(10):837-51.
19. Leventhal H, Phillips LA, Burns E. The Common-Sense Model of Self-Regulation (CSM): a dynamic framework for understanding illness self-management. J Behav Med. 2016;39(6):935-46.
20. Goossens NJ, Flokstra-de Blok BM, van der Meulen GN, Botjes E, Burgerhof HG, Gupta RS, et al. Food allergy knowledge of parents - is ignorance bliss? Pediatr Allergy Immunol. 2013;24(6):567-73.
21. Gupta RS, Springston EE, Smith B, Kim JS, Pongracic JA, Wang X, et al. Food allergy knowledge, attitudes, and beliefs of parents with food-allergic children in the United States. Pediatr Allergy Immunol. 2010;21(6):927-34.
22. Khanna R, Shifrin N, Nektalova T, Goldenberg G. Diet and dermatology: Google search results for acne, psoriasis, and eczema. Cutis. 2018;102(1):44;6;8.
23. Teasdale E, Muller I, Sivyer K, Ghio D, Greenwell K, Wilczynska S, et al. Views and experiences of managing eczema: systematic review and thematic synthesis of qualitative studies. British Journal of Dermatology 2020; doi:10.1111/bjd.19299
24. Le Roux E, Powell K, Banks JP, Ridd MJ. GPs’ experiences of diagnosing and managing childhood eczema: a qualitative study in primary care. British Journal of General Practice. 2018;68(667):e73.
25. Nottingham Support Group for Carers of Children with Eczema. Information 2020 [Available from: http://www.nottinghameczema.org.uk/information/index.aspx.

For peer review only - http://bmjopen.bmj.com/site/about/guidelines.xhtml

For peer review only

For peer review only - http://bmjopen.bmj.com/site/about/guidelines.xhtml

BOX AND TABLES

Box 1 Topics explored in Parent and GP interviews

Parent Interview	GP interviews
• Beliefs about food allergy and their origin<br>• Perceived or experienced acceptability of allergy investigations, including skin prick tests<br>• Facilitators of and barriers to uptake of skin prick tests and dietary advice<br>• Worry or social difficulties related to food allergies<br>• Strategies used to manage their child's eczema, e.g. excluding foods	• Beliefs about food allergy testing<br>• Views of the acceptability of allergy tests to parents<br>• Facilitators of and barriers to uptake of allergy investigations (including blood and skin prick tests) and dietary advice in primary care

For peer review only

Table 1 Parent participant characteristics (n=21)

Parent Characteristics	Number of participants
**Trial arm allocation**	
Intervention	15
Comparator	6
**Child's POEM score**	
Mild/moderate (<17)	16
Severe (>17)	5
**Area deprivation score***	
Low	5
Medium	8
High	8
**SPT results**	
Negative	14
Positive	1
N/A Comparator	6
**Education Level**	
Degree or higher	13
Diploma	1
A-level	3
G.C.S.E	2
NVQ	2

***Index of Multiple Deprivation (14) based on home postcode**

Table 2 GP participant characteristics (n=11)

GP Characteristics	Number of participants
**Years' experience**	
0-5	3
6-10	3
11-15	4
16-21	0
21+	1
**Confidence in managing eczema***	
Low	0
Medium	5
High	6
**Practice deprivation**	
**score****	6
Low	1
Medium	4
High	

***Self-reported scale 1-10, low=1-3, medium=4-7, high=8-10**

****Index of Multiple Deprivation (14)**

For peer review only - http://bmjopen.bmj.com/site/about/guidelines.xhtml

TEST Interview Topic Guide – PARTICIPATING PARENT

1. Introduction

- Thank the interviewee, introduce self, re-state purpose of the interview and main points from the information sheet, answer any questions, outline structure of interview
- Discussion of how interview will be recorded and transcribed, right to withdrawal from interview, issues of confidentiality, anonymisation and informed consent – *if agree start audio recorder*
- Verbal consent
    - Do you agree to our conversation being audio recorded?
    - Do you know you are free to stop the interview at any point and you may skip questions you would prefer not to answer?
    - Do you understand that quotations from the interview may be used to illustrate our findings, but it will not be possible to trace who said them?

2. Background

- Age of child and general eczema history: how/when was eczema first diagnosed? Length time eczema been an issue, severity over time
- Explore eczema management: Where have they sought advice on managing eczema? Doctor, health nurse, alternative practitioner (outside NHS), internet including forums? Any investigations up to point of study? What treatments advised/tried to date?
- How many children? Do siblings have eczema? What about related conditions e.g. asthma, hay fever?
- Currently breastfeeding? Y/N

3. Beliefs about allergy testing and role of allergies in eczema

- What do they know about the origins/cause of eczema?
- Do they think their child's eczema is related to an allergy (food or other)? Why do they give the answer they do?
- What does the parent know about food allergies? Is the parent aware of distinctions between food allergy, food intolerance, local skin irritation?
- Have they sought advice about food allergy testing? Explore understanding of food allergy testing e.g. different types of tests, food exclusion
- Explore previous experiences of food allergy testing in the family:
    - Have their child/other family members had allergy testing or excluded certain foods? If yes, how/why did this come about? Where did they seek advice on allergy testing/food exclusion?
    - What sort of tests - skin prick test, blood test? Other forms of testing encountered? Was this at home or through the NHS/private (and reason for this)? *Note: child should not have had food allergy testing other than at home as inclusion criterion*
- In their opinion, why would any parent want their child to have a food allergy test? Why would any parent not want their child to have food allergy test? Explore views of both food elimination and reintroduction strategy and skin prick tests. Are there reasons specific to skin prick tests?
- What about them – would they allow their child to have it or not? Probe reasons why it is/is not acceptable (generally, and skin prick test more specifically)

For peer review only

- What does parent think about excluding certain foods from the child's diet? What might challenges be (if any)? Worry? Social difficulties (parties, eating out)? School? Family set up? Shared parenting? Is there anything which could be done to overcome challenges?

4. Views on the trial and experiences of participating

- How did you first hear about the study? (letter, approached HCP, poster, flyer etc.)
- Views on being asked to take part in the study. Initial thoughts? What did they think about initial invitation letter, flyer, name of study/logo? PIS, baseline visit?
- Understanding of the purpose of trial. Understanding of what's involved in taking part in the trial. What does the parent like about the study?
- Why did the parent choose to take part in study (motivations)? Any concerns? Have these been overcome? How, why?
- Breastfeeding. What advice do you/would you want about your own and the child's diet? Any concerns?

Select which of the below sets of questions to ask depending on whether shorter/longer time since randomisation:

- **Early in trial (shorter time since randomisation)**
    - Clarify with route taken since allocation to trial arm. What's happened, who have they been referred to?
    - Parent's expectations
    - Understanding of procedures/what they need(ed) to do
    - What are their views on what happens next?
    - What does the parent expect to happen next? Explore expectations of skin prick test and food avoidance (if intervention)
    - Does the parent anticipate sticking to which arm they have been allocated? Why/why not?
    - Is there anything the parent would change about the way they have been involved in the trial so far?
    - What is their experience of completing the study questionnaires? Probe length, ease of completion, difficulty understanding/answering any items, burden
    - Check whether skin prick test done at same time as allergy history questionnaire. If yes, does the parent think their answers to the allergy history were influenced by having the skin prick test done at same time?
    - Has the parent spoken with other parents about being involved in the trial? Explore answer

- **Later in trial (longer time since randomisation)**
    - Clarify route taken since allocation to trial arm. What's happened, who have they been referred to?
    - Explore experiences of visits and tests, and their opinion of these:
        - How understandable was the advice?
        - Was it presented in a format they wanted it in?
        - Could they remember what they had been advised?
        - Explore views of information they may have been given in between
    - Was parent happy with outcomes of visits, tests, treatment, control allocation. Any concerns?
    - Experiences of eczema management since allocation. What has the parent been doing? Is this in line with what was recommended as part of trial allocation (i.e. did they stick to what was recommended)? What has gone well? Have there been any challenges? Do you think you are likely to continue to follow the advice?

For peer review only

- Understandings of components of intervention – skin prick test and oral food challenge:
  - Any concerns? What did the parent expect to happen? Did what happen match expectations? If not, what were the differences? Any unexpected effects?
  - Was the information provided about the test/challenge adequate or not? Was anything missing?
  - How seriously did they take the results of the tests? Why? Did the test results lead to certain actions/behaviours? If child is an infant, were there any changes to breast-feeding practices because of the intervention?
- Have there been any changes in their attitudes and beliefs about allergy testing? If yes, what changes and why? What aspects of what they experienced made most difference? Probe: written information (which topics – emollients, food allergies?), verbal explanations from dietician, website, skin prick test etc
- Is there anything the parent would change about the way they have been involved in the trial?
- What was their experience of completing the study questionnaires? Probe length, ease of completion, difficulty understanding/answering any items, burden
- Has the parent spoken with other parents about being involved in the trial and the skin allergy testing? Explore answer
- How long they would be prepared to keep feeding back to the trial?

5. Any other issues

- Any other issues the participant would like to raise?

For peer review only

COREQ (COnsolidated criteria for REporting Qualitative research) Checklist

A checklist of items that should be included in reports of qualitative research. You must report the page number in your manuscript where you consider each of the items listed in this checklist. If you have not included this information, either revise your manuscript accordingly before submitting or note N/A.

Topic	Item No.	Guide Questions/Description	Reported on Page No.
**Domain 1: Research team and reflexivity**			
Personal characteristics			
Interviewer/facilitator	1	Which author/s conducted the interview or focus group?	5
Credentials	2	What were the researcher's credentials? E.g. PhD, MD	1
Occupation	3	What was their occupation at the time of the study?	5
Gender	4	Was the researcher male or female?	n/a
Experience and training	5	What experience or training did the researcher have?	5
Relationship with participants			
Relationship established	6	Was a relationship established prior to study commencement?	
Participant knowledge of the interviewer	7	What did the participants know about the researcher? e.g. personal goals, reasons for doing the research	
Interviewer characteristics	8	What characteristics were reported about the inter viewer/facilitator? e.g. Bias, assumptions, reasons and interests in the research topic	
**Domain 2: Study design**			
Theoretical framework			
Methodological orientation and Theory	9	What methodological orientation was stated to underpin the study? e.g. grounded theory, discourse analysis, ethnography, phenomenology, content analysis	
Participant selection			
Sampling	10	How were participants selected? e.g. purposive, convenience, consecutive, snowball	5
Method of approach	11	How were participants approached? e.g. face-to-face, telephone, mail, email	5
Sample size	12	How many participants were in the study?	6
Non-participation	13	How many people refused to participate or dropped out? Reasons?	
Setting			
Setting of data collection	14	Where was the data collected? e.g. home, clinic, workplace	5
Presence of non-participants	15	Was anyone else present besides the participants and researchers?	
Description of sample	16	What are the important characteristics of the sample? e.g. demographic data, date	6
Data collection			
Interview guide	17	Were questions, prompts, guides provided by the authors? Was it pilot tested?	5
Repeat interviews	18	Were repeat inter views carried out? If yes, how many?	
Audio/visual recording	19	Did the research use audio or visual recording to collect the data?	6
Field notes	20	Were field notes made during and/or after the inter view or focus group?	
Duration	21	What was the duration of the inter views or focus group?	
Data saturation	22	Was data saturation discussed?	5
Transcripts returned	23	Were transcripts returned to participants for comment and/or	

For peer review only

Topic	Item No.	Guide Questions/Description	Reported on Page No.
		correction?	
**Domain 3: analysis and findings**			
Data analysis			
Number of data coders	24	How many data coders coded the data?	6
Description of the coding tree	25	Did authors provide a description of the coding tree?	
Derivation of themes	26	Were themes identified in advance or derived from the data?	6
Software	27	What software, if applicable, was used to manage the data?	6
Participant checking	28	Did participants provide feedback on the findings?	
Reporting			
Quotations presented	29	Were participant quotations presented to illustrate the themes/findings? Was each quotation identified? e.g. participant number	6-13
Data and findings consistent	30	Was there consistency between the data presented and the findings?	6-14
Clarity of major themes	31	Were major themes clearly presented in the findings?	6-13
Clarity of minor themes	32	Is there a description of diverse cases or discussion of minor themes?	

Developed from: Tong A, Sainsbury P, Craig J. Consolidated criteria for reporting qualitative research (COREQ): a 32-item checklist for interviews and focus groups. *International Journal for Quality in Health Care*. 2007. Volume 19, Number 6: pp. 349 – 357

Once you have completed this checklist, please save a copy and upload it as part of your submission. DO NOT include this checklist as part of the main manuscript document. It must be uploaded as a separate file.

For peer review only
